# Supplementary material for: Radiofrequency Combined with Intratumoral Immunotherapy: Preclinical Results and Safety in Metastatic Colorectal Carcinoma
Source: Pharmaceutics. 2024 Feb 23;16(3):315. doi: 10.3390/pharmaceutics16030315 (PMC10974839; doi:10.3390/pharmaceutics16030315)
Supplement: Supplementary file 1 [file pharmaceutics-16-00315-s001.zip › pharmaceutics-2853818-supplementary.pdf]

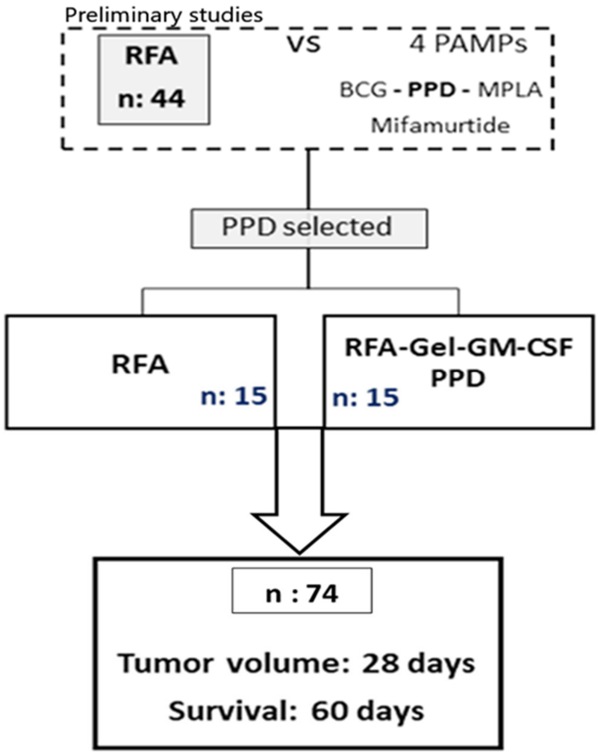

Figure S1. Flowchart of mice experiments. Sample size of groups of mice used for validation of the PPD vs. other 3 PAMPs candidate: RFA (n=44) vs. RFA-gels GMCSF-PPD (n=20) vs. RFA-gel-GMCSF- BCG (n=25) vs RFA-gel GMCSF-mifamurtide (Mepact n=24) vs RFA-gel-Monophosphoryl lipid A (MPLA (n=4). In the preliminary screening of three PAMP candidates for BCG replacement, only RFA-gel-GMCSF-PPD provided complete tumor regression. This formulation was selected for BCG replacement. To minimize the effects of variability in the volume and recurrence of the primary treated tumor, all groups of mice treated by standardized RFA- (n =44) in this preliminary experiment were used in the final study.

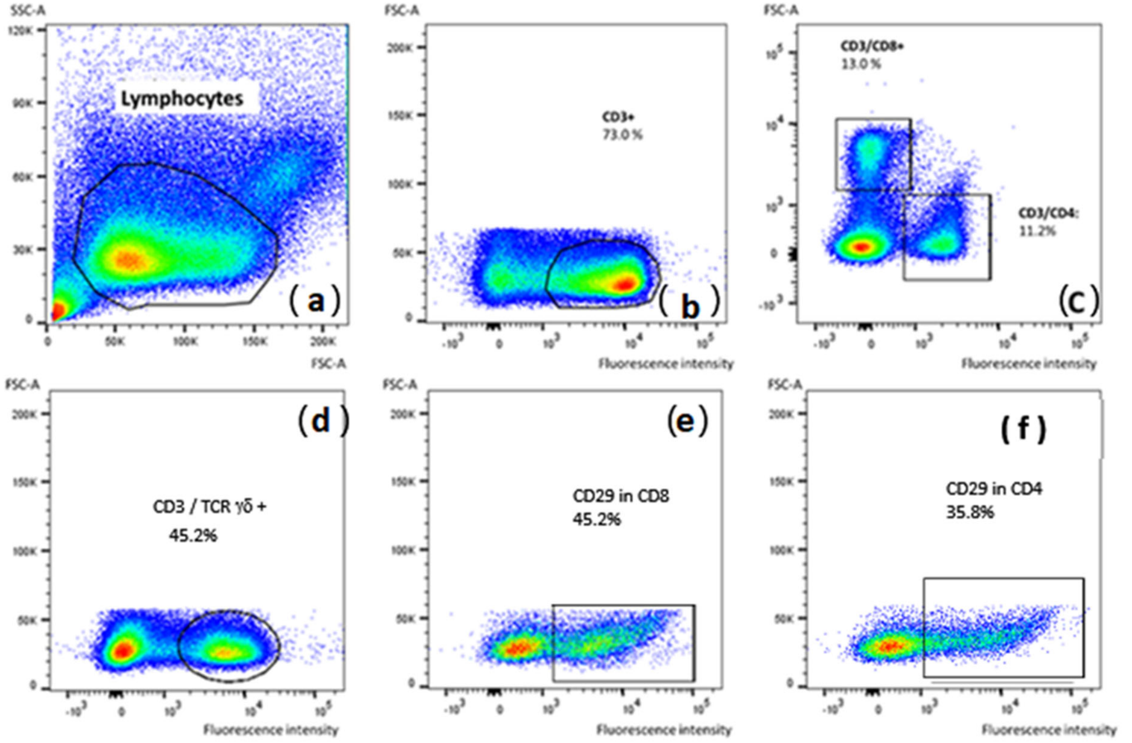

Figure S2. Flow cytometry analysis of the BMDC. Example of proportions in the cytoPlot is given for days 15 in pig 4; (**a**) CytoPlot of the gating strategy for (**b**) CD3; (**c**) CD3/CD8$^+$ and CD3/CD4$^+$ cells; (**d**) CD3/TCR ɣδ$^+$ cell; (**e**) CD8/CD29$^+$ cells and (**f**) CD4/CD29$^+$ cells.
